# The fate of the Arctic seaweed *Fucus distichus* under climate change: an ecological niche modeling approach

**DOI:** 10.1002/ece3.2001

**Published:** 2016-02-16

**Authors:** Alexander Jueterbock, Irina Smolina, James A. Coyer, Galice Hoarau

**Affiliations:** ^1^ Faculty of Biosciences and AquacultureNord UniversityUniversitetsalleen 118049BodøNorway; ^2^ Shoals Marine LaboratoryUniversity of New HampshireDurhamNew Hampshire03824USA

**Keywords:** Arctic ecosystem, cold‐temperate, competition, hybridization, intertidal macroalgae, key species, rocky intertidal

## Abstract

Rising temperatures are predicted to melt all perennial ice cover in the Arctic by the end of this century, thus opening up suitable habitat for temperate and subarctic species. Canopy‐forming seaweeds provide an ideal system to predict the potential impact of climate‐change on rocky‐shore ecosystems, given their direct dependence on temperature and their key role in the ecological system. Our primary objective was to predict the climate‐change induced range‐shift of *Fucus distichus*, the dominant canopy‐forming macroalga in the Arctic and subarctic rocky intertidal. More specifically, we asked: which Arctic/subarctic and cold‐temperate shores of the northern hemisphere will display the greatest distributional change of *F*. *distichus* and how will this affect niche overlap with seaweeds from temperate regions? We used the program MAXENT to develop correlative ecological niche models with dominant range‐limiting factors and 169 occurrence records. Using three climate‐change scenarios, we projected habitat suitability of *F*. *distichus* – and its niche overlap with three dominant temperate macroalgae – until year 2200. Maximum sea surface temperature was identified as the most important factor in limiting the fundamental niche of *F*. *distichus*. Rising temperatures were predicted to have low impact on the species' southern distribution limits, but to shift its northern distribution limits poleward into the high Arctic. In cold‐temperate to subarctic regions, new areas of niche overlap were predicted between *F*. *distichus* and intertidal macroalgae immigrating from the south. While climate‐change threatens intertidal seaweeds in warm‐temperate regions, seaweed meadows will likely flourish in the Arctic intertidal. Although this enriches biodiversity and opens up new seaweed‐harvesting grounds, it will also trigger unpredictable changes in the structure and functioning of the Arctic intertidal ecosystem.

## Introduction

Anthropogenic climate change, occurring faster than changes in the past 65 million years (Diffenbaugh and Field 2013), defines an ecological turning point: numerous species extinctions and poleward range shifts disturb species interactions and ecosystem services on a global scale (Brierley and Kingsford 2009; Barnosky et al. 2011). Throughout the 21st century, Arctic temperatures are predicted to rise twice as fast (>6°C until 2100, A1B SREC scenario) as the global mean temperature (Nakicenovic and Swart 2000; Meehl et al. 2007). As a consequence, the melting perennial Arctic ice cover will open up suitable habitat for temperate and subarctic species from the south (Boe et al. 2009). Subarctic isotherms already have shifted poleward up to seven times faster in the ocean than on land (Burrows et al. 2011).

Accordingly, marine species tracked rising temperatures by an order of magnitude more rapidly than terrestrial species (Parmesan and Yohe 2003; Poloczanska et al. 2013). Marine intertidal species are particularly sensitive to rising temperatures as they often exist at their upper temperature tolerance limits (Tomanek 2010). Thus, poleward shifts of intertidal species can serve as early warning signal of ecosystem‐level changes due to climate‐change.

Canopy‐forming seaweeds provide an ideal system to predict the impact of climate change on rocky shore ecosystems, because: (1) seaweed distribution depends directly on temperature isotherms (Breeman 1990; Jueterbock et al. 2013); and (2) seaweed species are foundational ‘ecosystem engineers’ (sensu Jones et al. 1994), providing food, habitat, and protection for a diverse range of species in the intertidal (Carss and Elston 2003; Christie et al. 2009; Harley et al. 2012). Marine macroalgae are also important carbon sinks, sequestering worldwide up to 0.46–2.55 pg (1 pg = 10^12^ kg) of carbon year^−1^ (reviewed in Mineur et al. 2015).

Seaweed diversity is highest in temperate regions, which extend in the northern hemisphere from the 9–10°C summer SST (sea surface temperature) isotherm in the north to the 20°C to 23°C winter SST isotherm in the south (van den Hoek 1975; Lüning et al. 1990). The southern edge of temperate seaweeds already has reacted to climate change, particularly in the Atlantic Ocean, which has warmed faster (Lee et al. 2011) and to greater depths (Barnett et al. 2005) than the Pacific or Indian Oceans. For example, the southern distribution limit of the brown macroalga *Fucus vesiculosus* has retreated northward from Southern Morocco (West Africa) by approximately 1200 km over the past 30 years (Nicastro et al. 2013). Disappearing seaweed meadows from warm‐temperate regions are often replaced by more stress‐resistant, but structurally less diverse crustose and foliose turf algae as well as calcified organisms like barnacles, mussels, and snails (Bartsch et al. 2012; Harley et al. 2012; Brodie et al. 2014). Therefore, poleward shifts of canopy‐forming seaweeds can trigger profound changes in the diversity and functioning of temperate rocky shore communities (Harley et al. 2012; Brodie et al. 2014; Mineur et al. 2015). The northern edge of temperate seaweeds is predicted to extend into Arctic regions that will be ice‐free within the next century (Jueterbock et al. 2013) and the poleward shift of the subtidal kelp species *Laminaria hyperborea* from northern Norway to the southern shores of Spitsbergen (Müller et al. 2009) supports the prediction.

**Figure 1:**
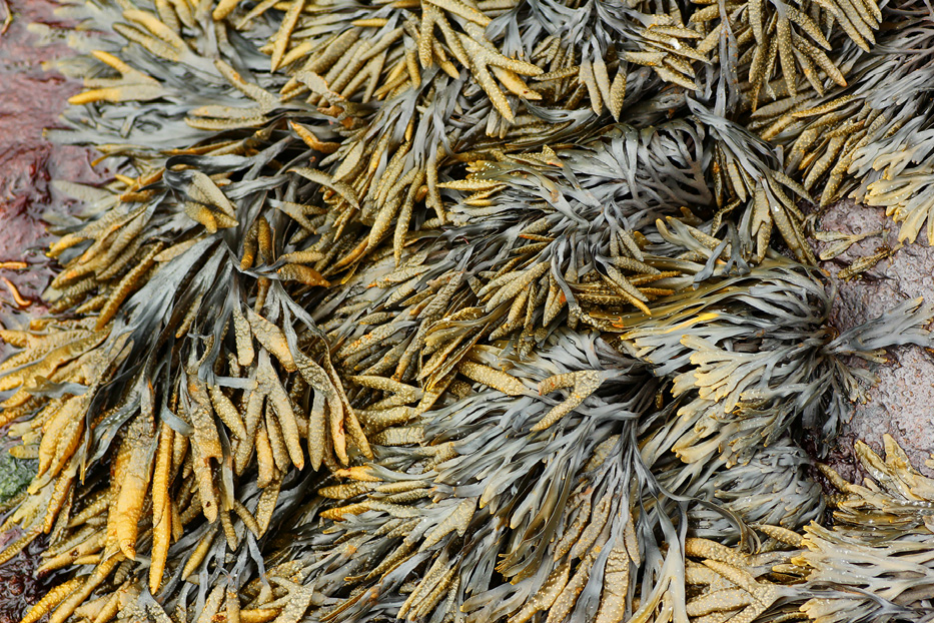
The canopy‐forming macroalga *Fucus distichus*.

The Arctic intertidal is poor in seaweed diversity and endemism (van den Hoek 1975; Lüning et al. 1990) and is dominated by the hermaphroditic brown macroalga *F*. *distichus* (L.) Powell 1957 (Fig. 1) (Coyer et al. 2011). The genus *Fucus* originated in the North Pacific and radiated in the North Atlantic after the opening of the Bering Strait into lineage 1 including *F. spiralis* and *F. vesiculosus*, and lineage 2 including *F. distichus* and *F. serratus* (Coyer et al. 2006a). The latter two species hybridize in Iceland and the Kattegat Sea, 150–200 years old secondary contact zones (Coyer et al. 2002, 2006b; Hoarau et al. 2015). *F*. *distichus* is the only species of its genus that is adapted to the Arctic, surviving several months under the ice (Svendsen et al. 2002). Along Arctic and sub‐Arctic coasts of both the North Atlantic and Pacific oceans, it split into several subspecies, each morphologically distinct but polyphyletic (Coyer et al. 2006a; Kucera and Saunders 2008; Laughinghouse et al. 2015). *F*. *distichus* is also likely to be locally adapted to the Arctic and subarctic as its response to elevated temperatures differed between populations from Svalbard and Kirkenes, Norway (Smolina et al. 2016). Where Arctic shores are protected from intensive spring ice scour, the species complex *F*. *distichus* dominates the intertidal (Lüning et al. 1990; Becker et al. 2009; Wiencke and Amsler 2012), supporting diverse and unique ecological communities (Ellis and Wilce 1961; Munda 2004).

Thus, range shifts of the dominant *F*. *distichus* may correlate with climate change‐induced displacement of the Arctic and cold temperate phytogeographic regions (Bartsch et al. 2012; Wiencke and Amsler 2012; Brodie et al. 2014) and are likely to have strong consequences for seaweed‐harvesting, ecosystem functioning and biodiversity in the northern hemisphere. Therefore, the sensitivity of the Arctic intertidal ecosystem to climate change largely depends on how far the distribution limits of *F*. *distichus* will shift in response to rising temperatures.

Rising temperatures are expected to extend the northern distribution of *F*. *distichus* into the high Arctic (Coyer et al. 2011), but to leave its southern distribution limit unaffected (Hiscock et al. 2004). Over the past century, human transport extended the native distribution of *F*. *distichus* on the E‐Atlantic coast >400 km southward despite rising temperatures (reviewed in Forslund 2009). Instead of temperature, photoperiod, which regulates receptacle formation and emryonic development in *F*. *distichus* (McLachlan 1974; Edelstein and McLachlan 1975; Bird and McLachlan 1976), was suggested as potential range‐limiting factor to the south (Hiscock et al. 2004).

Our primary objective was to develop correlative Ecological Niche Models with dominant range‐limiting factors to predict range shifts of *F*. *distichus* in the Arctic and Subarctic intertidal in response to global climate change. Using three climate change scenarios, we asked: Which rocky shores of the North Atlantic, Arctic, and North Pacific will display the greatest distributional change of *F*. *distichus* and how will this affect niche overlap with seaweeds from temperate regions?

## Materials and Methods

Correlative Niche Modeling, also called Ecological Niche Modeling (Elith and Leathwick 2009), is a powerful approach that has been widely used to predict distributional range shifts under climate change (e.g. Perry et al. 2005; Wiens et al. 2009; Alahuhta et al. 2011). Correlating the geographic distribution of a species with environmental conditions that are involved in setting its geographic range limits allows to identify the species’ realized niche (reviewed in Guisan and Zimmermann 2000; Elith and Leathwick 2009). This niche is then projected into geographic space as mapped values of habitat suitability, which can be converted to binary values by applying a threshold sharply discriminating suitable from nonsuitable habitat. The projected habitat suitability is used to evaluate the fit of the model to the species' occurrence records. Finally, to project the species' geographic habitat suitability into the future, present‐day temperatures are replaced by temperatures predicted under CO_2_ emission scenarios.

### Occurrence records

Several ecomorphs are found within *F*. *distichus*, each morphologically distinct and polyphyletic (Coyer et al. 2006a; Laughinghouse et al. 2015). Since these ecomorphs may interbreed and are not supported by any species concept (Laughinghouse et al. 2015), we synonymize *F*. *distichus* (L.) Powell 1957 with *F*. *gardneri* P.C. Silva 1953 and three of the subspecies previously united by Powell (1957): subsp. *distichus* (Linnaeus) Powell 1957; *edentatus* (De La Pylaie) Powell 1957; and *evanescens* (C. Agardh) Powell 1957;. However, *F*. *distichus* subsp. *anceps* (Harvey et Ward ex Carruthers) Powell 1957; a dwarf form found in wave‐exposed areas of the high intertidal, may be a separate genetic entity (based on microsatellite data, Coyer et al. 2011) and is, thus, excluded from the *F*. *distichus* species complex.

**Figure 2:**
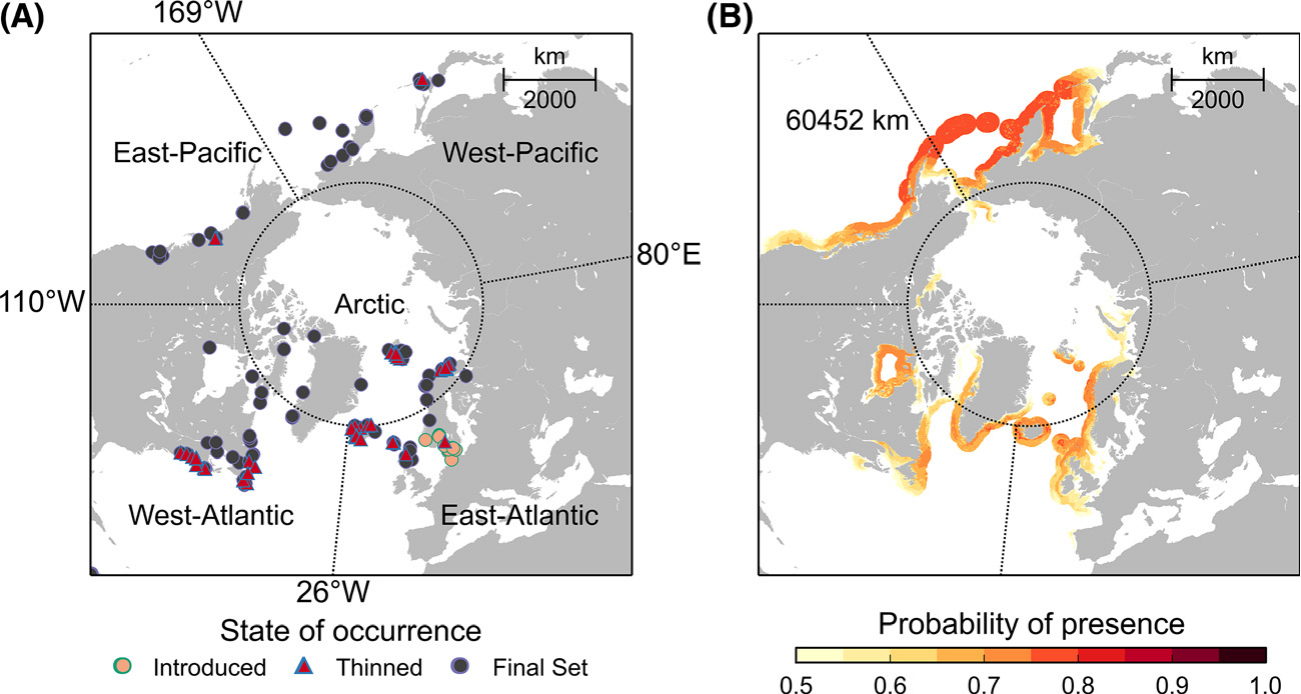
Occurrence records (A) and projected present‐day habitat suitability (B) of *Fucus distichus* (lambert azimuthal equal‐area projection). Longitudinal and latitudinal border lines delimit five geographic regions (West‐ and East‐Atlantic, West‐ and East‐Pacific, and the Arctic (north of the polar circle at 66°N, see Table 1). (A) To avoid sampling‐bias, 72 locations (red triangles) in areas of dense sampling effort were filtered out. Locations at which *F*. *distichus* was recently introduced were removed (indicated by orange points). The 97 locations that were used for Niche Modeling are shown as blue points; (B) habitat suitability of coastal regions is shown in gradients of logistic probabilities of presence (0.5–1). Probabilities below the probability threshold of 0.5 were considered unsuitable.

The 169 occurrence records of *F*. *distichus* (Fig. 2A, Table S1), from which ENMs (Ecological Niche Models) were trained to identify suitable habitat conditions, were based on personal observations and literature with detailed descriptions of the geographic location. This is the most comprehensive set of occurrence records for *F*. *distichus*, covering its entire range of distribution. Sites located inland were shifted to the closest coastal waters using the java program ‘moveCoordinatesToClosestDataPixel.jar' (Verbruggen 2012b).

We thinned the 169 occurrence sites in order to avoid the model from misinterpreting strong sampling effort with high habitat suitability (Phillips et al. 2009). Accordingly, we created kernel density grids of the occurrence records with the bkde2D function of the R package ‘KernSmooth’ v 2.23‐13 (Wand 2014) (using a bandwidth of 3.0 in longitudinal and 1.5 in latitudinal direction). These grids informed the java program ‘Occurrence Thinner’ v.1.04 (Verbruggen 2012a) about areas of high local densities from which it randomly removed 72 occurrence sites (using thresholds t1 = 0.2 and t2 = 1.0) (Fig. 2A, Table S1).

### Background points

Environmental conditions in the distributional range of *F*. *distichus* were captured by 50,000 background sites placed randomly along the coastline between 25°N and 84°N with the R package ‘raster’ (Hijmans 2015). Shores of Arctic Russia, Canada, and Alaska were excluded, as the lack of occurrence records would be interpreted as unsuitable habitat (Fig. S1). While we expected that *F*. *distichus* occurs in these regions, they are simply too remote and inaccessible to allow census of marine macroalgae.

### Environmental variables

We considered an initial set of 26 environmental variables (Table S2) representing various candidate predictors that are potentially relevant for the distribution of macroal‐gae. Twenty‐four of these were downloaded from the Bio‐ORACLE database (http://www.oracle.ugent.be/index.html, real values): four surface air temperature derivatives (mean, minimum, maximum, and range), representing the aerial ‘weather’ impact on the intertidal, were compiled and described in Jueterbock et al. (2013); the other 20 Bio‐ORACLE variables, oceanographic data measured from the water column, in Tyberghein et al. (2012). To identify the importance of seasonality in photoperiod for the distribution of *F*. *distichus*, we compiled two global rasters with the R packages ‘raster’ (Hijmans 2015) and ‘insol’ (Corripio 2014): (1) Summer solstice, representing the hours of daylight at midsummer (21 Jun); and (2) Winter solstice, representing the hours of daylight at the shortest day of the year (21 Dec).

### Model selection

Habitat suitability for *F*. *distichus* under present‐day and future environmental conditions was estimated using correlative Ecological Niche Models (ENMs) compiled with the program MAXENT v3.3.3e (Phillips and Dudík 2008). To avoid over‐fitting the models to the occurrence records, we reduced the set of environmental variables in a stepwise fashion with the R package ‘MaxentVariableSelection’ (Jueterbock 2015). We compiled an initial MAXENT model with all 26 variables and excluded those variables with a relative contribution score <5%. In the retained set of variables, we removed those variables that were correlated (correlation coefficient, Pearson's *r*, >0.9 or <−0.9, based on the 50,000 background locations) with the variable of highest contribution. The remaining set of variables was used to compile a new MAXENT model. Again, variables with contribution scores <5% were removed and remaining variables that were correlated with the variable of second‐highest contribution were discarded. This process was repeated until left with a set of uncorrelated variables that all had a model contribution >5%.

After each step, we assessed the model performance based on the sample‐size‐adjusted AICc (Akaike information criterion) (Akaike 1974) (based on code in ENMTools (Warren et al. 2010)) and the area under the receiver operating characteristic (AUC) estimated from test data (Fielding and Bell 1997). While AICc values were estimated from single models that included all occurrence sites, AUC values were averaged over 10 replicate runs which differed in the set of 50% test data that were randomly subsampled from the occurrence sites and withheld from model construction. Since models selected by AUC as performance estimator can over‐predict a species' realized niche and fail to recognize its fundamental niche (Jiménez‐Valverde 2012), we selected the model of lowest AICc value, which is expected to be better transferable to future climate scenarios (Warren and Seifert 2011; Warren et al. 2014). In addition, model‐overfitting was estimated by the difference between AUC values from test and training data (AUC.Diff) (Warren and Seifert 2011). We performed variable selection for a range of *β* values from 0 to 15 in increments of 0.5.

Equilibrium with the environment is one of the main assumptions of ENMs (Elith et al. 2010) but it is unclear whether this assumption holds at the 19 occurrence sites to which *F*. *distichus* was introduced between the late 19th and early 20th century: Bergen (Hoarau et al. 2015), the Oslofjord (Bokn and Lein 1978; Simmons 1989), and the Kattegat Sea/western Baltic (Hylmö 1933; Schueller and Peters 1994; Wikström et al. 2002). We performed variable selection with and without the 19 non‐native occurrences sites (Fig. 2A, Table S1).

### Present‐day and future habitat suitability predictions

The model of highest performance (lowest AICc, Table S3) identified the optimal beta‐multiplier as well as the optimal sets of occurrence sites and environmental variables, which were used to project habitat suitability of *F*. *distichus* under present‐day and future conditions. The projections were based on logistic output grids averaged over the 10 replicate MAXENT models. All models were run with hinge features only.

Future predictions differed from the present‐day prediction only in the maximum SST, which was projected to future conditions by three CO_2_ emission scenarios: B1 (low emissions), A1B (medium emissions) and A2 (high emissions) (Nakicenovic and Swart 2000). Grids that projected maximum SST to years 2100 (all three scenarios) and 2200 (scenarios B1 and A1B) were compiled by Jueterbock et al. (2013) and downloaded from http://www.oracle.ugent.be/download. We applied a threshold of 0.5, which best reflected the species' contemporary distribution limits, to convert the logistic model outputs to binary grids that sharply discriminate suitable from nonsuitable habitat.

### Length of suitable coastline and niche overlap with temperate seaweeds

From the binary projections, we calculated the length of coastline providing suitable habitat (separately for five coastal regions, Table 1) by taking the square root of the approximated surface area (calculated with the *area* function of the ‘raster’ R package, Hijmans 2015) that was reflected by pixels directly adjacent to the coastline (obtained with the *boundaries* function of the ‘raster’ package). For the same five coastal regions, we estimated the niche overlap between *F*. *distichus* and the temperate seaweed species *F*. *vesiculosus*,* F*. *serratus*, and *Ascophyllum nododsum* with the similarity statistic *I* (introduced in Warren et al. 2008) that was calculated with the *nicheOverlap* function of the ‘dismo’ R package (Hijmans et al. 2015). Niche models for the three temperate species were compiled by Jueterbock et al. (2013).

**Table 1:** Latitudinal and longitudinal boundaries that were used to define five oceanic regions within the distributional range of *Fucus distichus*

Region	Western boundary	Eastern boundary	Northern boundary	Southern boundary
West‐Atlantic	110°W	26°W	66°N	–
East‐Atlantic	26°W	80°E	66°N	–
West‐Pacific	80°E	169°W	66°N	–
East‐Pacific	169°W	110°W	66°N	–
Arctic	–	–	–	66°N

## Results

### Model performance and importance of environmental variables

The model of lowest AICc (2712, Figs. S2, S3) was built with: only native occurrence sites, a beta‐multiplier of 2, and four uncorrelated environmental variables with a model contribution >4.4% (Table S3): maximum SST, and the concentrations of calcite, nitrate and chlorophyll a. Maximum SST was the most important variable (57.85% model contribution, Table S3) in discriminating suitable from nonsuitable habitat for *F*. *distichus,* confirming that temperature is generally the most important range‐limiting factor of seaweeds (Breeman 1988, 1990; Bartsch et al. 2012; Jueterbock et al. 2013). Calcite concentration had a model contribution of 24.08%, followed by nitrate concentration with a contribution of 13.67% and chlorophyll concentration (4.40%, Table S3). Minimum surface air temperature had a variable contribution >5%, but was removed from the model because it correlated significantly with maximum SST (Table S3). Habitat suitability was predicted to be highest from 5°C to 15°C maximum SST, and was positively correlated with the concentration of nitrate, an important nutrient for *F*. *distichus* (e.g. Rueter and Robinson 1986), but negatively correlated with the concentrations of calcite and chlorophyll (Fig. S4). Calcite and chlorophyll a may be only indirectly relevant or correlated with more relevant environmental factors not included in the model. Although highly speculative, calcite, one of two polymorphs of calcium carbonate, could favor crustose coralline algae and calcified herbivores such as snails and sea urchins, thus increasing interspecific competition with and grazing pressure on *F*. *distichus* (Harley et al. 2012). Chlorophyll a is positively correlated with water turbidity caused by any kind of autotrophic biomass in shallow water, and thus, may be negatively correlated with light availability.

The average AUC.Test of the present‐day model (based on 10 replicate runs) was 0.83, suggesting that the model could well‐discriminate between presence and absence sites, Fig. S2). The low AUC.Diff value (0.02, Table S3) indicates that the model was not overfit to the occurrence locations and, thus, was well‐transferable to future climate conditions (Warren and Seifert 2011).

Habitat suitability was highest in the Pacific region (Fig. 2B) with hot‐spots of suitable conditions along the Aleutian Islands and the eastern coast of the Kamchatka Peninsula. Projected and realized southern distribution limits matched well along west coasts of both the Atlantic (Cape Cod, 43°N) and the Pacific Oceans (Hokkaido, 42°N) (Fig. 2B, Table S1). On the east coasts of both oceans, however, habitat suitability was projected 13° to 19° further south than southernmost occurrence records.

North of the polar circle (66°N) suitable habitat projections matched occurrence records on the shores of the Faroe Islands and the southwestern coast of Svalbard (Fig. 2B). Unfavorable temperatures (<5°C) were the main reason for low habitat suitability in the Russian and Canadian Arctic, as well as in NE‐Canada. In addition, calcite concentrations >0.02 mol m^−3^ lowered habitat suitability in the Khatanga Gulf and in the East Siberian Sea in Arctic Russia, as well as in the Beaufort Sea and along the Alaskan Bering Sea coast.

### Future projections of habitat suitability and niche overlap with temperate species

**Figure 3:**
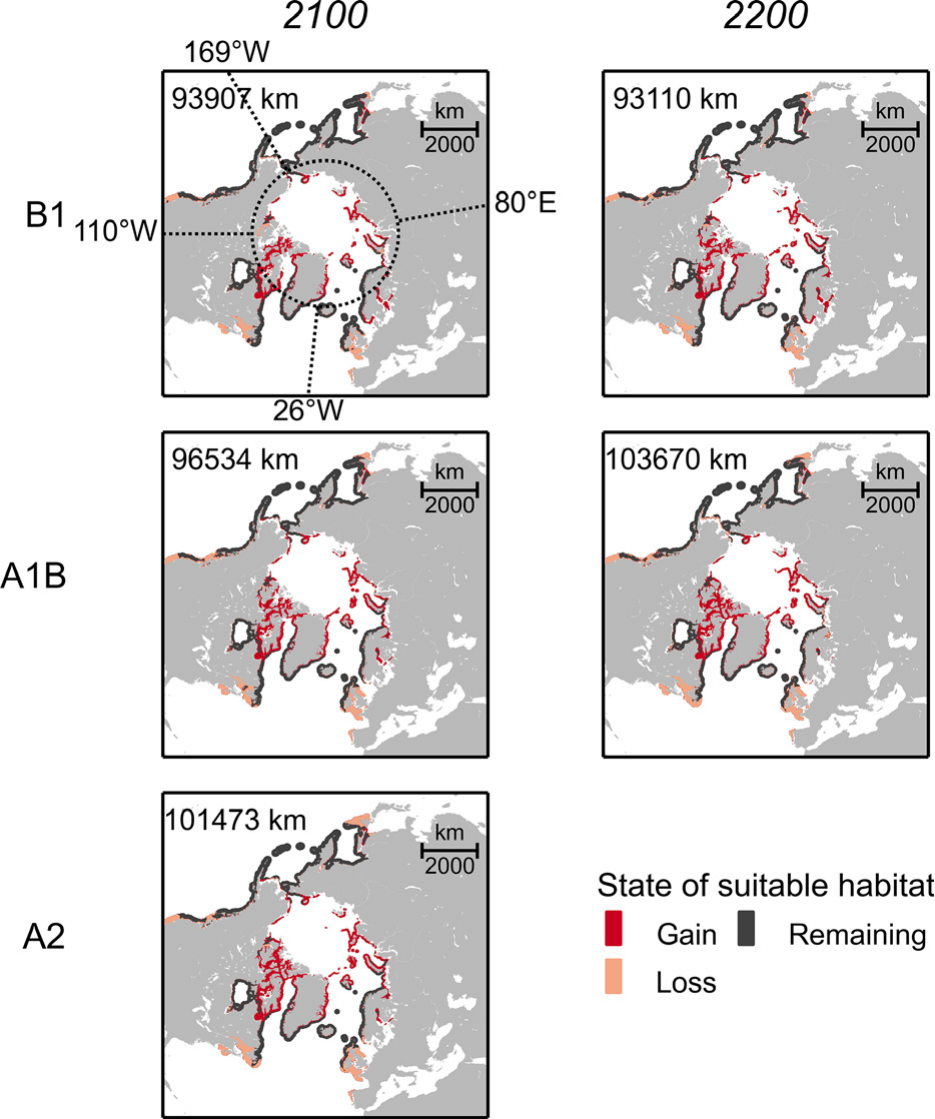
Habitat suitability changes of *Fucus distichus* until year 2100 and 2200 (compared to present‐day conditions) under the SREC scenarios B1 (low emission), A1B (medium emission), and A2 (high emission) (lambert azimuthal equal‐area projection). Coastal areas with logistic probabilities >0.5 were regarded suitable and are included in the estimated length of suitable coastline (in km); estimated lengths of suitable coastline for each of five geographic regions (delimited by the latitudinal and longitudinal border lines, specified in Fig. 2A) are given in Table S4.

The length of suitable coastline was predicted to increase from 60,452 km under present‐day conditions (Fig. 2B) to >93,000 km by 2100 under the weakest emission scenario B1 (Fig. 3). Most drastic changes were projected in the high Arctic region (north of 66°N) where suitable habitat was predicted to expand up to 60,000 km until 2200 (emission scenario A1B; Table S4). Loss of habitat along the East‐Atlantic and ‐Pacific coasts spanned mostly unoccupied regions of the fundamental niche of *F*. *distichus* (Fig. 3). On the West‐Atlantic coast, all emission scenarios predicted unfavorably warm temperatures south of Newfoundland by 2100. On the West‐Pacific coast, only short stretches of coastline were predicted to become unsuitable along the coast of Hokkaido.

**Figure 4:**
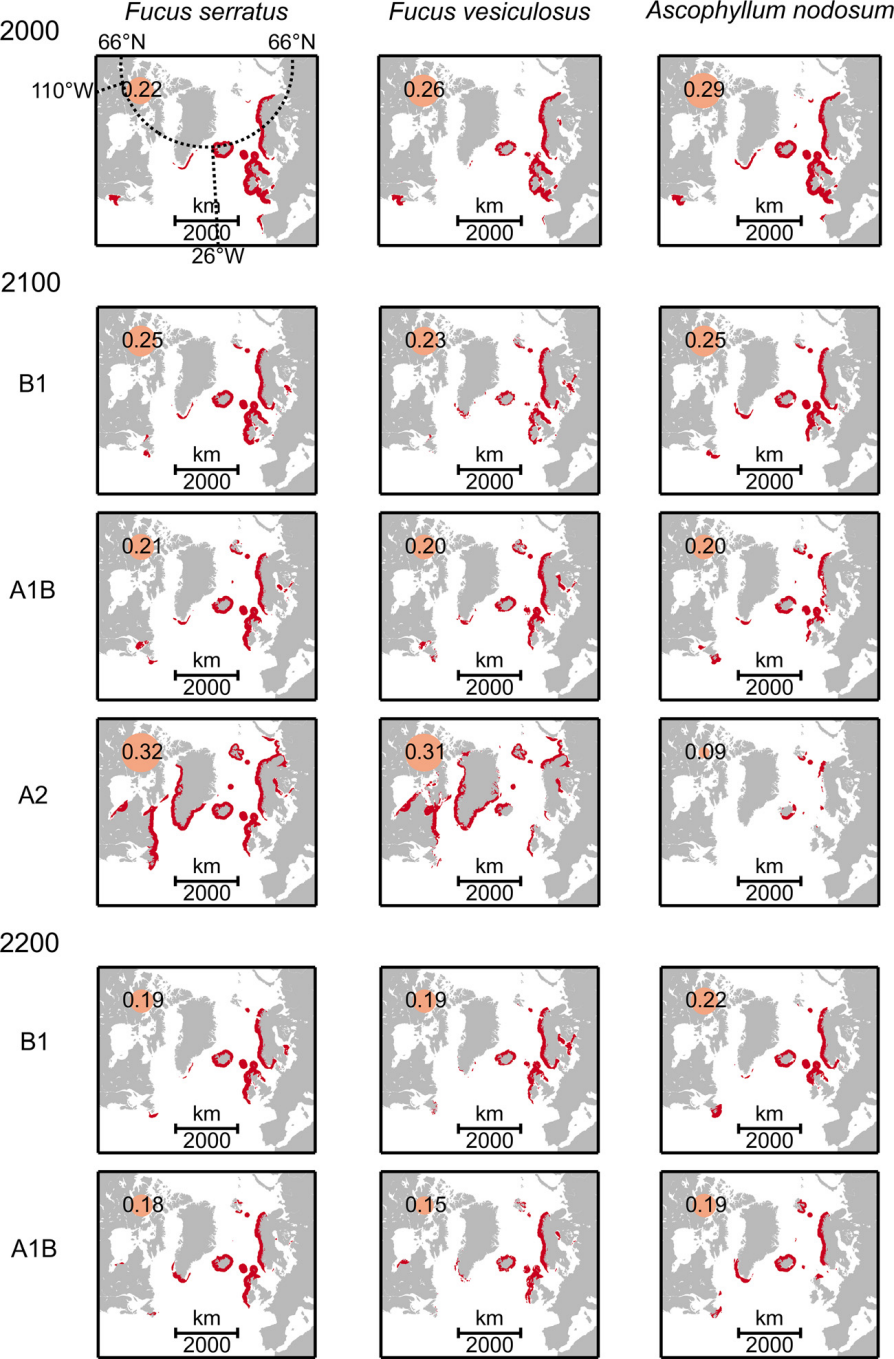
Projected niche overlap of *Fucus distichus* with three temperate macroalgae under present‐day (year 2000) and future (year 2100 and 2200) conditions (lambert azimuthal equal‐area projection). Projections are shown for three emission scenarios: B1 (low emission), A1B (intermediate emission), and A2 (high emission). Overall niche identities *I* (Warren et al. 2008) are provided (potential range 0–1) and comparatively visualized by the proportion of the yellow circles. Estimated niche identities for each of five geographic regions (delimited by the latitudinal and longitudinal border lines, specified in Fig. 2A) are given in Table S5. Niche models for the three temperate species were compiled by Jueterbock et al. (2013).

Overlap between the fundamental niches of *F*. *distichus* and the niches of the three temperate fucoid seaweeds (*F*. *serratus*,* F*. *vesiculosus,* and *A*. *nodosum*) was predicted to shrink, despite predictions that distributions of all species will shift polewards (Fig. 4, Table S5). Current niche identities in the Atlantic (>20%) were predicted to fall below 20% by year 2200 (except the overlap with *A*. *nodosum* under emission scenario B1). New regions of overlap were predicted along the shores of Svalbard, southern Greenland, and Newfoundland (Fig. 4). Under emission scenario A2, projected extension of *F*. *serratus* and *F*. *vesiculosus* into Arctic Russia, Canada, as well as Greenland by 2100, increased niche overlap with *F*. *distichus* by >30%.

## Discussion

### Distribution limits and shift on west coasts

The predicted and realized southern range limits of *F*. *distichus* on the West‐Pacific and West‐Atlantic coasts coincided with summer SST isotherms >20°C (Table S6). Here, temperature was likely the direct range‐limiting factor, because 20°C was empirically identified as the upper temperature limit for normal development of embryos (McLachlan 1974), the most temperature‐sensitive life stage in brown algae (e.g. Nielsen et al. 2014).

Along the west coasts of both oceans, the predicted poleward shift was small, possibly because of the close proximity of summer SST isotherms. In agreement with a faster increase of Atlantic than Pacific surface temperatures (Lee et al. 2011), the southern limit of *F*. *distichus* was predicted to shift only 1°N on the West‐Pacific coast (except in scenario A2) but 3°N on the West‐Atlantic coast.

### Distribution limits and shift on east coasts

Along the east coasts of both oceans, the predicted fundamental niche of *F*. *distichus* reached further south than its present distribution limit. Indeed, because the prevailing southerly long shore currents induce upwelling off California and North Spain, the east coasts are thermally suitable to lower latitudes than the west coasts. The mismatch between fundamental and realized niches was expected, given that human transport could extend the native distribution of *F*. *distichus* >400 km southward over the past century, despite rising temperatures (reviewed in Forslund 2009). Thus, factors other than temperature may limit the native southern distribution of *F*. *distichus* on the East‐Atlantic coast.

Photoperiod is an important factor regulating seaweed reproduction (Dring 1988) and interactive effects between photoperiod and temperature may influence the distribution of *F*. *distichus*. Receptacle formation is restricted to the winter‐months between autumn and spring equinox (Bird and McLachlan 1976). After the spring equinox, embryos develop normally where temperatures remain <20°C, but become aberrant >20°C and >12 h daylight (McLachlan 1974). However, these boundaries cannot explain why the realized southern distribution limits of *F*. *distichus* are located north of 50°N on the east coasts although covariation between temperature and photoperiod should be favorable for reproduction and embryonic development to at least the same latitudes as on the west coasts (42°–43°N). Accordingly, our variable selection considered photoperiodic seasonality as an irrelevant factor in defining distribution limits (Table S3). We believe that repeated mismatches between the southern distribution limits and the embryos' upper thermal tolerance limits on the east coasts of both ocean basins are better explained by repeated environmental restrictions than by local ecotypic variation (Smolina I., University of Nordland, submitted manuscript).

Interspecific competition may at least partly explain the southern distribution limits of *F*. *distichus*; in combination with high air temperatures, on the East‐Atlantic coast, and in combination with nitrogen depletion, on the East‐Atlantic and ‐Pacific coasts. Nitrate, the prevailing source of nitrogen for macroalgae in seawater, was identified as an important range‐limiting factor for *F*. *distichus* (13.57% model contribution, Table S3), with average concentrations of 4–5 *μ*mol L^−1^ at the realized southern distribution edges, but only 1–2 *μ*mol L^−1^ at the predicted southern edges on the east coasts (Table S6). Decreased nitrogen supply in combination with a threshold concentration for uptake of ammonium – the only nitrogen source during air exposure – is a competitive disadvantage (Thomas et al. 1985) and might lead to competitive exclusion of *F*. *distichus* south of its realized distribution boundaries on the east coasts. Indeed, in the Kattegat and Western Baltic Sea, where *F*. *distichus* was introduced in the last century (Hylmö 1933; Schueller and Peters 1994; Wikström et al. 2002), it is mostly confined to nutrient‐enriched waters such as harbors, where other fucoids are scarce (Wikström et al. 2002).

Moreover, a shift of the upper zonation limit of *F*. *distichus* from mid‐tide level on Iceland (Munda 2004) and in the Canadian Arctic (Ellis and Wilce 1961) to −0.25 m below low‐water level in the Oslofjord region (Bokn et al. 1992) may indicate avoidance of high air temperatures in its southern range, as reported for *F*. *serratus* (Pearson et al. 2009). In our analysis, surface air temperature played a relevant role for setting distribution limits of *F*. *distichus*, but was excluded from the model because it was correlated with maximum SST (Table S3). In the lower subtidal where *F*. *distichus* could avoid high air temperatures, however, it is generally outcompeted by its sister species *F*. *serratus* (Ingólfsson 2008; Johnson et al. 2012). Consequently, *F*. *serratus* could set the southern distribution limit of *F*. *distichus* on the East‐Atlantic coast, by preventing it from escaping hot air temperatures in the shallow subtidal zone. On the East‐Pacific coast, *F*. *distichus* is unlikely to be excluded from the intertidal zone as *F*. *serratus* is absent and maximum surface air temperatures of 19°C at the predicted southern distribution limit are 4°C below the maximum air temperatures at the realized southern distribution limit on the West‐Pacific coast (Table S6).

Our future models predicted no change in the realized southern distribution limit of *F*. *distichus* on the east coasts of both oceans (Fig. 3). This is not surprising, given that the maximum SST of 14–15°C at both southern distribution edges on the east coasts were 4–7°C lower than on the west coasts (19–21°C, Table S6). Accordingly, only the unfilled fundamental niches were predicted to shift northwards, thereby increasing the fit between fundamental and realized niche limits in future. This means that any further southward extension, such as the 400 km southward range extension along East‐Atlantic coast over the past century (Forslund 2009), becomes increasingly difficult (Fig. 3).

### Colonization of Arctic regions

In the Arctic, the length of suitable coastline for *F*. *distichus* was predicted to at least triple from 12,000 to 43,000 km by 2100 (Table S4). However, since bottom‐substrate was not included as environmental factor in our niche models, this prediction overestimates the length of suitable coastline in the Russian Arctic that is mainly characterized by unsuitable soft‐bottom substrate (Widdowson 1971; as quoted in Müller et al. 2010). Increasing coastal erosion and river sediment discharge due to melting sea ice, rising sea levels, and melting permafrost (Syvitski 2002; ACIA, 2004; Macdonald et al. 2015) will likely reduce rocky coastline that provides today suitable hard‐bottom substrate along the Canadian Arctic as well as Arctic islands off the Russian mainland, the Kola Peninsula, Spitsbergen, and Greenland (Müller et al. 2009).

Colonization of remaining hard‐bottom substrates requires effective dispersal. On one hand, dispersal of *F*. *distichus* is limited, because: (1) fucoid zygotes generally settle <10 m from the egg‐bearing female (Serrão et al. 1997; Dudgeon et al. 2001); (2) fucoid populations can be genetically differentiated by as few as 2 km (Coyer et al. 2003); and (3) natural dispersal rates may not exceed 0.2–0.6 km year^−1^ (Coyer et al. 2006b; Brawley et al. 2009). On the other hand, however, temperatures <5°C can delay zygote attachment and thus increase the dispersal capacity of *F*. *distichus* (Coleman and Brawley 2005). *F*. *distichus* is a hermaphrodite with frequent self‐fertilization and rafting thalli can be an effective means of long‐range dispersal (Thiel and Gutow 2005), since only one fertile individual is necessary to establish a new population at distant sites. In the near future, dispersal of *F*. *distichus* into Arctic regions may also increase as a result of increased shipping along ice‐free routes in the Canadian and Russian Arctic (Lasserre and Pelletier 2011).

While perennial ice‐cover is predicted to disappear entirely by 2100 (Boe et al. 2009), seasonal ice cover may persist in some areas. Nevertheless, the presence of sea ice now or in the future is unlikely to hinder *F*. *distichus* from colonizing much of the Arctic. Many northern rocky shores are characterized by boulders and cracks/crevices, in which small individuals are protected from scouring ice (Adey and Hayek 2005). On Svalbard, *F*. *distichus* survives several months under the ice and frequently is exposed to freezing temperatures during low tide (Svendsen et al. 2002). Given that the UV‐B filtering stratospheric ozone layer is most rapidly depleting in polar areas (Karsten et al. 2009), UV‐radiation may restrict *F*. *distichus* to the lower intertidal or subtidal zone by preventing embryonic development in the upper intertidal (Schoenwaelder et al. 2003).

### Changes in diversity and biotic interactions

Since most Arctic macroalgae are subtidal, immigration of the canopy‐forming *F*. *distichus* will likely enrich biological diversity in the high Arctic intertidal. Indeed, intertidal seaweed cover and biomass on Svalbard increased with rising temperatures from 1988 to 2008 (Węsławski et al. 2011). Biological diversity will likely also increase in the subarctic intertidal where temperate seaweeds, which are associated with a rich community of epiphytic algae and free‐living invertebrates (Wikström and Kausky 2004), were predicted to immigrate from the south (Jueterbock et al. 2013; Brodie et al. 2014).

While gametic incompatibility separates *F*. *distichus* reproductively from its sister species *F*. *serratus* in 10,000 year‐old sympatry zones (i.e. northern Norway) (Hoarau et al. 2015), the two sister species interbreed in Iceland and the Kattegat Sea, where *F*. *serratus* was introduced in the last 150–200 years (Coyer et al. 2002, 2006b; Hoarau et al. 2015). Although regions of niche overlap between the two sister species are predicted to decrease, new hybrid zones may form along the shores of Svalbard, S‐Greenland and N‐Canada, where both species find suitable habitat in the future (Fig. 4). Most likely, prezygotic isolation barriers will arise in these future contact zones, since negative selection of hybrids was observed in all previously established contact zones (Coyer et al. 2002; Hoarau et al. 2015).

In southern Sweden, where *F*. *distichus* invaded in 1924 (Hylmö 1933), it is restricted to sites where native fucoids are scarce (Wikström et al. 2002), suggesting competitive inferiority to temperate seaweeds. In its native range, however, *F*. *distichus* can co‐occur with the dominant temperate macroalgae *F*. *vesiculosus*,* F*. *serratus*, and *A*. *nodosum* on the same shore albeit at slightly different zonation levels (Munda 2004; Coyer et al. 2006b). In the Arctic, *F*. *distichus* may have a competitive advantage because of its adaptation to cold conditions and long dark periods. For example, reduced temperature (7°C vs. 17°C) lowered the competitive power of the ephemeral alga *Ulva compressa* (formerly known as *Enteromorpha compressa)* on germling settlement and growth of *F*. *distichus* (60% vs. 100% yield reduction) (Steen 2004). Although temperate species may not outcompete *F*. *distichus* in the Arctic, they may truncate its upper and lower zonation boundaries. In Iceland and Nova Scotia, for example, introduced *F*. *serratus* replaced *F*. *distichus* in the lower intertidal (Ingólfsson 2008; Johnson et al. 2012).

Our models projected highest habitat suitability for *F*. *distichus* in the northwestern Pacific region, which is likely the origin of most of the Arctic flora, including the seaweed genus *Fucus* (Adey et al. 2008). Our habitat suitability projections agree with a deep branching haplotype network for Pacific samples (Laughinghouse et al. 2015), both suggesting stable suitable conditions in both glacial and interglacial periods. The ancestral core population of *F*. *distichus*, however, is likely centered in the Canadian Arctic (based on a haplotype network of a mtDNA intergenic spacer) and is moved to northwestern Atlantic and Pacific regions during glacial periods (Laughinghouse et al. 2015). The predicted northward extension of *F*. *distichus* suggests that the Canadian Arctic intertidal will again become a central region of genetic exchange and dilution between the Atlantic and Pacific peripheral subspecies (Coyer et al. 2011) (Laughinghouse et al. 2015).

## Conclusion

Our Niche Models predict that rising temperatures will barely affect the southern distribution limits of *F*. *distichus*. This is due to the close proximity of summer isotherms along the west coasts and because other factors than temperature set the southern distribution limit of *F*. *distichus* on the east coasts of both the Pacific and the Atlantic Oceans. In the Arctic region, however, rising temperatures will largely increase suitable habitat for *F*. *distichus* and other canopy‐forming macroalgae from temperate regions. We conclude that rising temperatures, while threatening seaweed meadows in warm‐temperate regions, will foster seaweed meadows in the Arctic intertidal. Although this enriches biodiversity and opens up new seaweed‐harvesting grounds, it will also trigger unpredictable changes in the structure and functioning of the Arctic intertidal ecosystem.

## Conflict of Interest

None declared.

## Data Accessibility

Global ASCII grids with daylength values during summer and winter solstice and ASCII grids representing habitat suitability of *Fucus distichus* and its niche overlap with temperate macroalgae under present‐day and future (2100, 2200) conditions are available from the Pangea database: http://doi.pangaea.de/10.1594/PANGAEA.848687.

The R package ‘MaxentVariableSelection’ is available on CRAN (https://cran.r-_project.org/web/packages/MaxentVariableSelection).

## Supporting information


**Figure S1.** Background (pseudo‐absence) sites.
**Figure S2.** Performance of niche models measured by AUC.Test values.
**Figure S3.** Performance of niche models measured by AICc values.
**Figure S4.** Response curves of the four environmental variables that were identified as most important range‐limiting factors.

**Table S1.** Occurrence records of *Fucus distichus*.
**Table S2.** Sources of grids of the 26 environmental variables.
**Table S3.** Variable selection process.
**Table S4.** Suitable coastlengths for *F*. *distichus*.
**Table S5.** Niche identities between *F*. *distichus*/and three temperate macroalgal species.
**Table S6.** Environmental conditions at distribution edges of *F*. *distichus*.
