# Supplementary material for: The fate of the Arctic seaweed Fucus distichus under climate change: an ecological niche modeling approach
Source: Ecol Evol. 2016 Feb 16;6(6):1712–24. doi: 10.1002/ece3.2001 (PMC4801954; doi:10.1002/ece3.2001)
Supplement: Supplementary file 1 — Figure S1. Background (pseudo‐absence) sites. Figure S2. Performance of niche models measured by AUC.Test values. Figure S3. Performance of niche models measured by AICc values. Figure S4. Response curves of the four environmental variables that were identified as most important range‐limiting factors. [file ECE3-6-1712-s001.doc]

*Ecology and Evolution*

**SUPPORTING INFORMATION - FIGURES**

**The fate of the Arctic seaweed *Fucus distichus* under climate change: an ecological niche modeling approach**

Jueterbock A., Smolina I., Coyer J.A. and Hoarau G.

**Figure S1:** Background (pseudo-absence) sites.

**Figure S2**: Performance of niche models measured by AUC.Test values.

**Figure S3**: Performance of niche models measured AICc values.

**Figure S4**: Response curves of the four environmental variables that were identified as most important range-limiting factors.


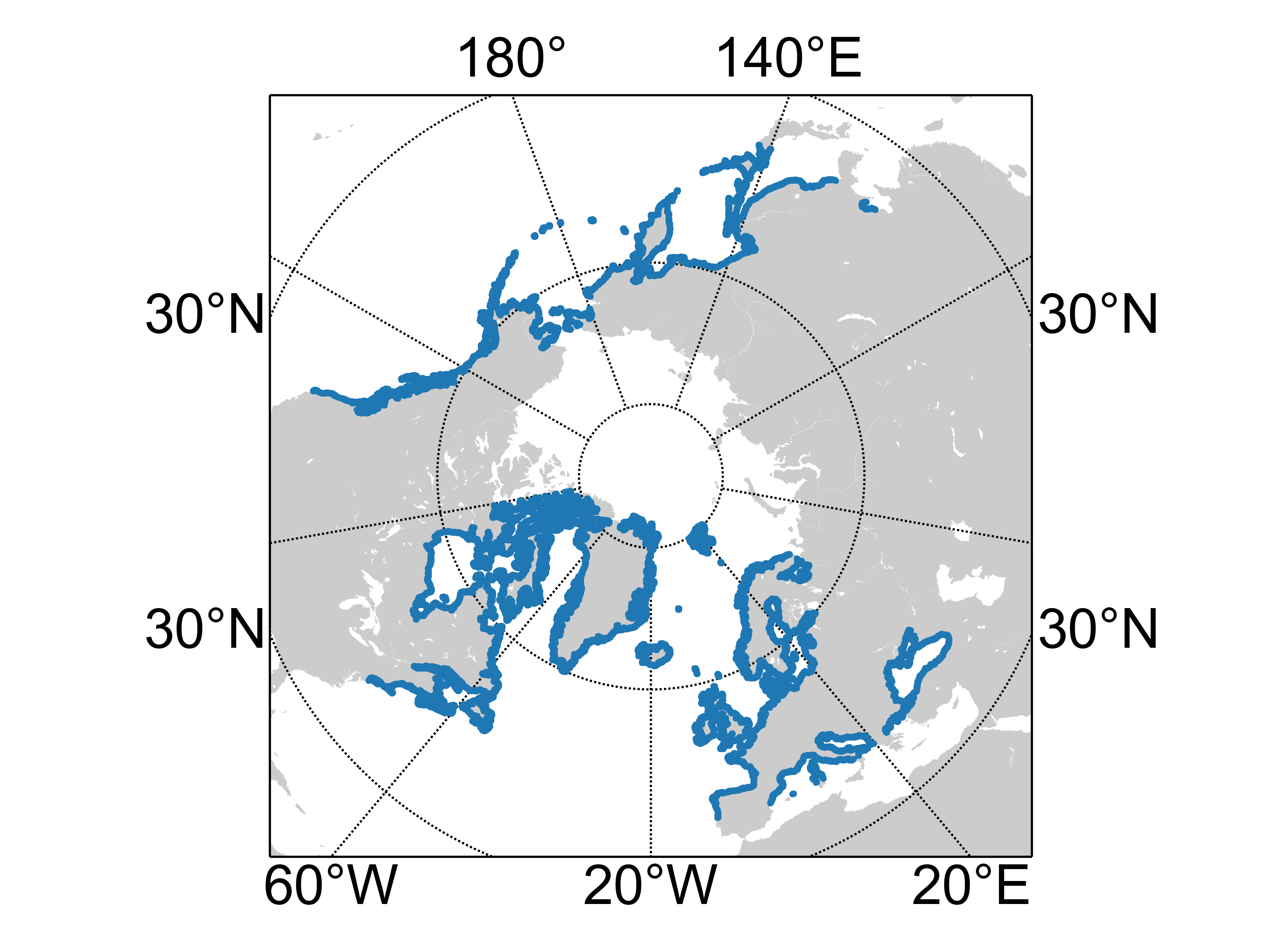


**Figure S1** Coastal background (pseudo-absence) sites providing information on environmental conditions within the latitudinal range of distribution of *F*. *distichus*.


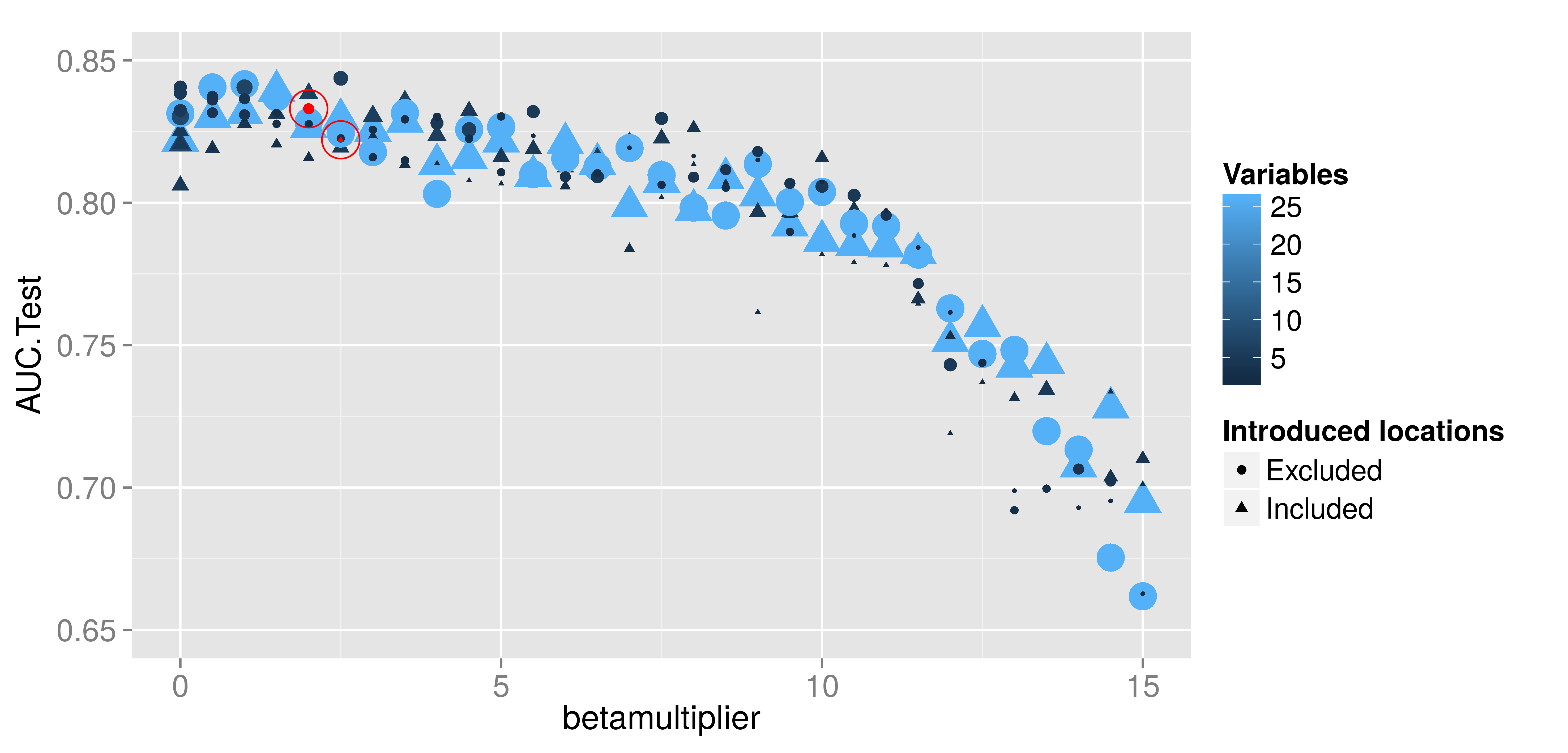


**Figure S2** Comparison of the performance of niche models that differed in the set of occurrence sites (including versus excluding locations where *Fucus distichus* was introduced) and the beta-multiplier (0 to 15 with increments of 0.5). Model-performance was estimated by the area under the receiver operating characteristic estimated from test data (AUC.Test) (Fielding & Bell, 1997). Each combination of beta-multiplier and set of occurrence sites started with 26 variables and was simplified in a stepwise fashion by removing those variables with low contribution-scores (<5%) and high correlation with other variables (correlation coefficients >0.9 or <-0.9). The number of variables in each model is encoded both by color and size. The models of highest performance (based on AICc (Akaike, 1974), not AUC.Test) are marked in red for both sets of occurrence sites.


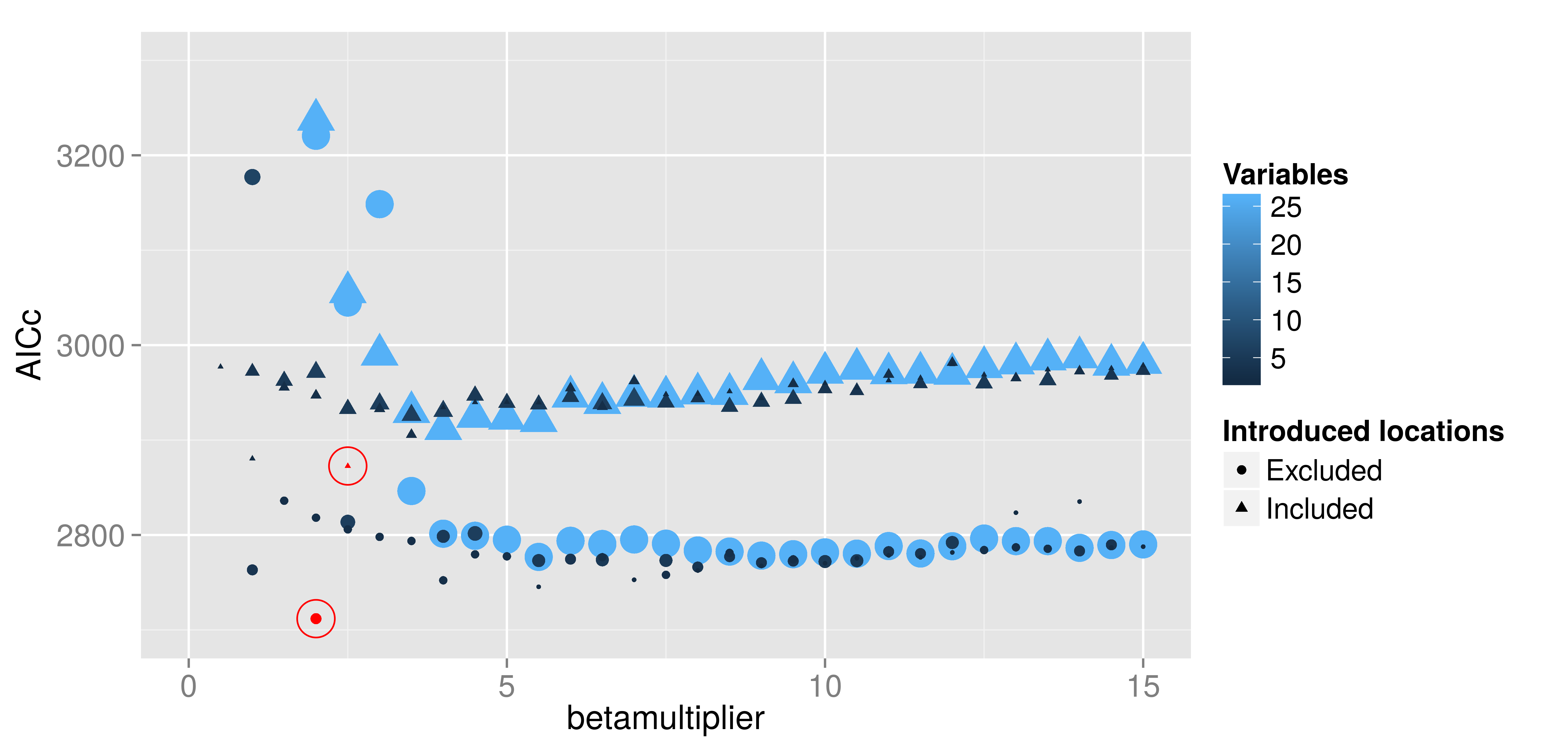


**Figure S3** Comparison of the performance of niche models that differed in the set of occurrence sites (including versus excluding locations where *Fucus distichus* was introduced) and the beta-multiplier (0 to 15 with increments of 0.5). Model-performance was estimated by the sample-size-adjusted Akaike information criterion (AICc) (Akaike, 1974). AICc values could not be calculated where the number of model parameters exceeded the number of occurrence sites. Each combination of beta-multiplier and set of occurrence sites started with 26 variables and was simplified in a stepwise fashion by removing those variables with low contribution-scores (<5%) and high correlation with other variables (correlation coefficients >0.9 or <-0.9). The number of variables in each model is encoded both by color and size. The models of highest performance (lowest AICc) are marked in red for both sets of occurrence sites.


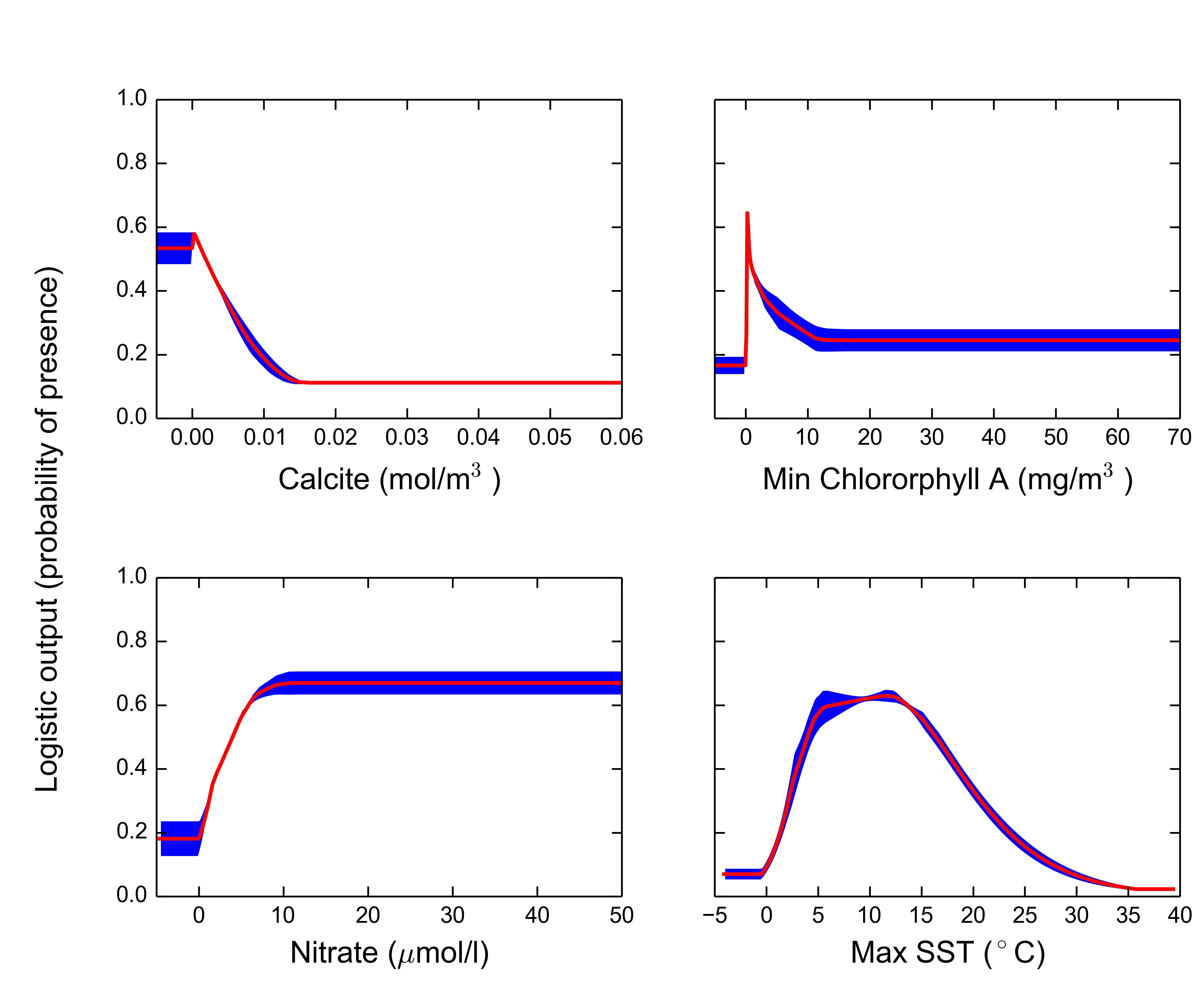


**Figure S4** Response curves of the four environmental variables that were chosen for niche modelling (see Table S3). The curves show how the mean (in red) logistic probability of presence of *F*. *distichus* depends on each of the four variables over ten replicate models; the range of two standard deviations are shown as blue shade. SST: Sea Surface Temperature.

**References**

Akaike, H. (1974) A new look at the statistical model identification. *IEEE Transactions*

*on Automatic Control* , **19**, 716–723.

Fielding, A.H. & Bell, J.F. (1997) A review of methods for the assessment of prediction errors in conservation presence/absence models. *Environmental Conservation*, **24**,

38–49.
